# Trends in carbapenemase-producing Enterobacteriaceae, France, 2012 to 2014

**DOI:** 10.2807/1560-7917.ES.2017.22.6.30461

**Published:** 2017-02-09

**Authors:** Laurent Dortet, Gaëlle Cuzon, Valérie Ponties, Patrice Nordmann

**Affiliations:** 1 Associated National Reference Center for Antibiotic Resistance, Le Kremlin-Bicêtre, France; 2 Faculty of Medecine, South-Paris University, Le Kremlin-Bicêtre, France; 3 Bacteriology-Hygiene Unit, Bicêtre Hospital, Assistance Publique / Hôpitaux de Paris, Le Kremlin-Bicêtre, France; 4 Pasteur Institute, Ecology and evolution of resistance to antibiotics unit, Paris, France; 5 The French Public Health Agency, Santé Publique France, Saint-Maurice, France; 6 Emerging Antibiotic Resistance Unit, Medical and Molecular Microbiology, Department of Medicine, University of Fribourg, Fribourg, Switzerland; 7 European INSERM Laboratory (LEA, IAME), Medical and Molecular Microbiology, University of Fribourg, Fribourg, Switzerland; 8 University of Lausanne, Institute for Microbiology, University hospital Center, Lausanne, Switzerland

**Keywords:** K. pneumoniae, OXA-48, OXA-181, NDM, KPC, multidrug resistance, France

## Abstract

In 2014, a total of 2,976 Enterobacteriaceae isolates with decreased susceptibility to carbapenems were received at the French Associated National Reference Center for Antibiotic Resistance (NRC) and were characterised for their molecular resistance mechanism to carbapenems and compared with results obtained during 2012 and 2013.The overall number of enterobacterial isolates with decreased susceptibility to carbapenems received at the NRC rapidly increased (more than twofold in two years) with a growing proportion of carbapenemase producers (23.1% in 2012 vs 28.6% in 2013 vs 36.2% in 2014). Between 2012 and 2014, the main carbapenemase type was OXA-48, with an increase in OXA-48 variants (mostly OXA-181) and NDM producers, whereas the number KPC producers decreased. We identified a potential spread of OXA-181 producers in the tropical region of Africa. Finally, OXA-48 and OXA-48-related enzymes remained the predominant carbapenemases in France. The number of carbapenemase-producing *Escherischia coli* isolates was multiplied by fivefold between 2012 and 2014, suggesting a possible dissemination in the community.

## Introduction

During the last decade, Gram-negative isolates, in particular Enterobacteriaceae, with a decreased susceptibility to carbapenems have been increasingly reported in Europe [1,2]. In Enterobacteriaceae, decreased susceptibility to carbapenems may be due to (i) a beta-lactamase with significant hydrolytic activity towards carbapenems, i.e. a carbapenemase, or (ii) a combination of overexpression of beta-lactamases possessing a weak carbapenemase activity towards carbapenems, i.e. extended spectrum beta-lactamase and/or cephalosporinase, with a decreased outer-membrane permeability or efflux overexpression [1]. The most clinically-relevant carbapenemases encountered in Enterobacteriaceae belong to either Ambler class A (mostly KPC-type) [3], or Ambler class B (metallo-beta-lactamases (MBLs)) such as IMP-, VIM- and NDM-types) [1,4] or Ambler Class D (OXA-48-like enzymes) [5].

According to the results of the European Survey on Carbapenemase-producing Enterobacteriaceae (EUSCAPE) survey [6], four European countries (Greece, Italy, Malta, Turkey) are facing a situation where carbapenemase-producing Enterobacteriaceae (CPE) are endemic. However, endemicity is associated with different types of carbapenemases in different countries: in Greece VIM and KPC, in Italy KPC and in Malta and Turkey OXA-48. Although most European countries have reported an increase in the spread of CPE, once again, strong geographical differences exist in terms of the carbapenemase types involved. KPC producing Gram-negative bacteria were mostly reported in Italy and Greece. OXA-48 producers were more widespread in some western European countries (Belgium, France, Spain) and in Romania and Turkey in the eastern part of the continent. VIM producers were endemic in Greece and interregional spread has been described in Italy, Spain and Hungary. Finally, NDM-producing Enterobacteriaceae were found to be more prevalent in central and eastern Europe (e.g. Poland, Romania). A precise identification of carbapenemase production and type is important for (i) the follow up of the spread of carbapenemase producers (ii) the timely identification of outbreaks and their prevention and (iii) the choice of treatment with novel drugs such as ceftazidime/avibactam active against producers of Ambler class A and D but not on class B carbapenemases [7].

Here, we assessed the epidemiology of Enterobacteriaceae with decreased susceptibility to carbapenems in France and analysed its evolution between 2012 and 2014.

## Methods

### Specimen collection

**Figure 1:**
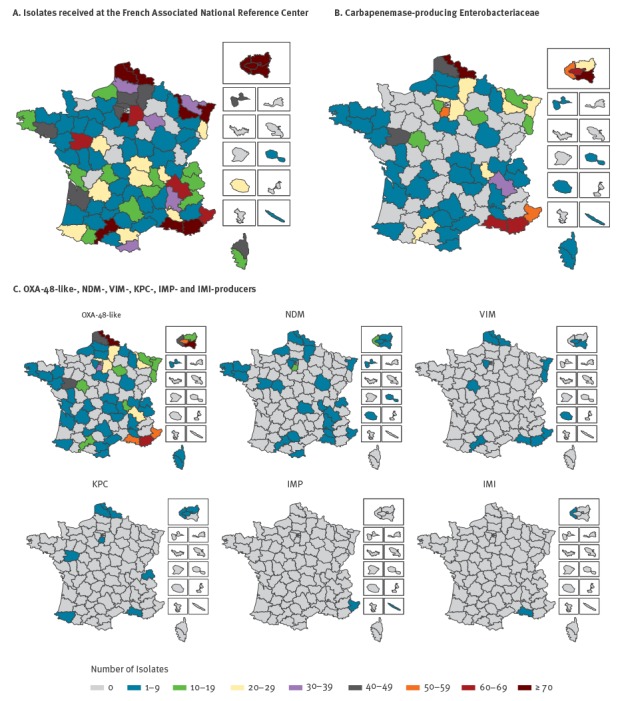
Geographic distribution of **A.** Isolates received at the French Associated National Reference Center for Antibiotic Resistance **B**. Carbapenemase-producing Enterobacteriaceae **C.** OXA-48-like-, NDM-, VIM-, KPC-, IMP- and IMI-producers, France, 2012–2014

From January 2012 to December 2014, 6,682 enterobacterial isolates (1,485 in 2012; 2,225 in 2013 and 2,972 in 2014) were received and tested for carbapenem activity at the French Associated National Reference Center for Antibiotic Resistance (NRC) in Le Kremlin-Bicêtre. Isolates were submitted from the whole of France, including French overseas territories. They were recovered from both clinical and screening specimens, and sent on a voluntary basis by any type of laboratory (n = 486) related to any health facility such as private and public hospitals, nursing homes, and community laboratories (Figure 1A).

Isolates with reduced susceptibility to carbapenems (ertapenem, meropenem, or imipenem) according to the Antibiogram Committee of the French Society of Microbiology (CA-SFM) (i.e. inhibition diameter < 22 mm, < 22 mm and < 25 mm for meropenem, imipenem or ertapenem respectively by disc diffusion) [8,9] were investigated for carbapenemase activity.

With each strain, provision of critical information was compulsory, such as the origin of specimens (screening rectal sample or any type of clinical sample), date of isolation, information regarding patient’s travel abroad in the year preceding the strain isolation (if yes, the country was recorded), the type of laboratory (hospital, community laboratory).

Duplicated isolates from the same patient were excluded from the study. If different species or different carbapenemase types were recovered from the same patient, the corresponding isolates were taken into consideration individually. Isolates were also re-identified at the NRC using a MALDI-TOF spectrometric technique (Maldi-Biotyper, Bruker Daltonique SA, Wissembourg, France). Most of them were *Klebsiella pneumoniae* (36%), *Enterobacter cloacae* (33.3%) and *Escherichia coli* (15%).

### Carbapenemase detection and molecular identification

The carbapenemase production was detected using the biochemical-based technique, the Carba NP test, as previously described [10]. Carbapenemase gene screening was performed by PCR aimed at identifying the *bla*_KPC_, *bla*_NDM_, *bla*_VIM_, *bla*_IMP_, *bla*_IMI_ and *bla*_OXA-48_-like genes [11]. In case of positive PCR, sequencing of the full-length gene was performed. A decreased susceptibility to carbapenems due to (i) an outer-membrane permeability defect, (ii) an overexpression of a cephalosporinase (chromosome-encoded or plasmid-acquired) associated with outer-membrane permeability defect, (iii) an extended spectrum beta-lactamase (ESBL) production associated with outer-membrane permeability defect or (iv) association of an ESBL and overexpression of a cephalosporinase outer-membrane permeability defect were suspected when the Carba NP test and PCR screening results were negative [12].

## Results

### Epidemiology of carbapenemase-producing Enterobacteriaceae

**Figure 2:**
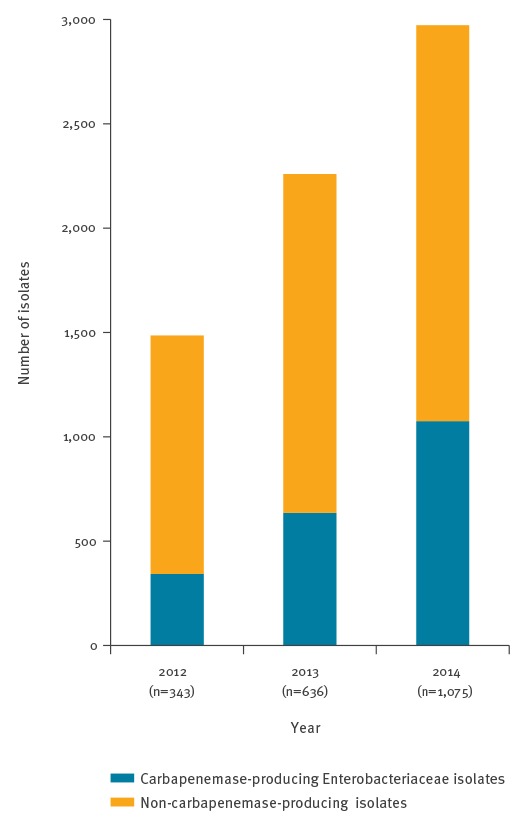
Distribution of carbapenemase-producing Enterobacteriaceae and non-carbapenemase-producing isolates by year, France, 2012–2014

According to EUCAST breakpoints [13], most of the isolates (99.6%, n = 6,655) received at the NRC were non-susceptible to at least one of the three carbapenems tested (imipenem, meropenem, ertapenem). The number of enterobacterial isolates with decreased susceptibility to carbapenems received at the NRC increased from 1,485 in 2012 to 2,225 in 2013 and 2,972 in 2014. The percentage of CPE among the Enterobacteriaceae with decreased susceptibility to carbapenems rose from 23.1% in 2012 to 28.6% in 2013 and 36.2% in 2014 (Figure 2) [12].

The number of carbapenemase-producing Enterobacteriaceae isolates identified is indicated below each year on the x-axis.

In 2014, carbapenemases were OXA-48- (85.6%), NDM- (8.5%), VIM- (2.7%), KPC- (1.8%), and IMI-like enzymes (0.3%) (Table 1).

**Table 1:** Distribution of carbapenemase types identified among carbapenemase-producing Enterobacteriaceae, France, 2014 (n = 1,075)

Type of carbapenemase	n	%
OXA-48-like	920	85.6
KPC	19	1.8
NDM	91	8.5
VIM	29	2.7
IMP	3	0.3
IMI	3	0.3
OXA-48-like + NDM	7	0.7
OXA-48-like + VIM	2	0.2
NDM + VIM	1	0.1
**Total**	**1,075**	**100**

**Figure 3:**
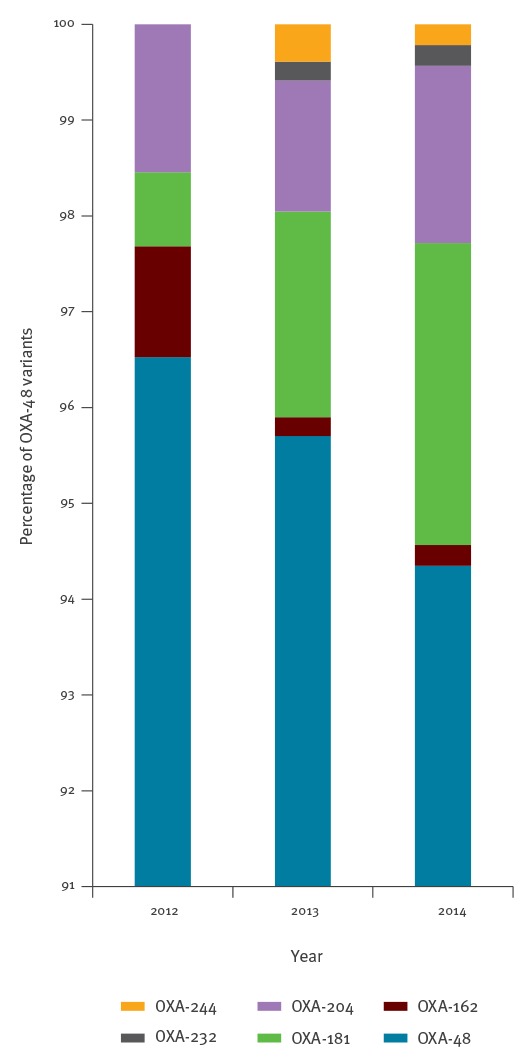
Distribution of OXA-48 variants among OXA-48-like carbapenemases by year, France, 2012–2014

From 2012 to 2014, carbapenemase producers were recovered from patients hospitalised and/or living in three main regions: the north, the south-east and the Paris area (Figure 1B), mostly following the global geographic distribution of OXA-48-like producers (Figure 1C). NDM producers seemed to be randomly scattered across France. Of note, all CPE, except one, recovered on Réunion island, a French overseas department and region in the Indian Ocean, were of the NDM type (Figure 1C). Finally, all CPE recovered in French New Caledonia were of the IMP type (Figure 1C). The number of OXA-48-like producers and NDM producers constantly increased from 2012 to 2014 (256, 512 and 920 OXA-48-like producers and 27, 61 and 91 NDM producers in 2012, 2013 and 2014, respectively). Contrary to this, the number of KPC producers decreased over the same period of time (39, 29 and 19 KPC producers in 2012, 2013 and 2014, respectively). One of the most relevant features observed between 2012 and 2014 is the increased diversity of OXA-48-like producers, which is mostly related to the identification of the OXA-181 variant (Figure 3).

In addition, in 2014, an OXA-48-like variant with decreased susceptibility to carbapenems but devoid of any carbapenemase activity, OXA-405 was evidenced [14].

As previously observed, most of the carbapenemase-producing Enterobacteriaceae obtained in 2014 were nosocomially acquired bacterial species, including *K. pneumoniae* (57.1%), *E. cloacae* (9.9%) and *Citrobacter freundii* (3.5%) (Table 2 and Table 3) [12].

**Table 2:** Distribution of carbapenemase-producing Enterobacteriaceae by bacterial species, France 2014 (n = 1,075)

Enterobacterial species	n	%
*Klebsiella pneumoniae*	614	57.1
*K. oxytoca*	19	1.8
*Escherichia coli*	256	23.8
*Enterobacter cloacae*	106	9.9
*E. aerogenes*	13	1.2
Other *Enterobacter* spp.	4	0.4
*Citrobacter freundii*	38	3.5
*C. koseri*	9	0.8
Other *Citrobacter* spp.	1	0.1
*Serratia* spp.	7	0.7
*Proteus mirabilis*	1	0.1
Other *Proteae* (*Proteus spp.. Providentia* spp.)	1	0.1
*Morganella morganii*	4	0.4
Other	2	0.2
**Total**	**1,075**	**100**

**Table 3:** Distribution of carbapenemase and non-carbapenemase-producing isolates by enterobacterial species, France, 2014

Enterobacterial species	Total number of isolates	Carbapenemase-producing Enterobacteriacease										Non-carbapenemase-producing Enterobacteriacease					
		CPE	OXA-48-like	KPC	NDM	VIM	IMP	IMI	OXA-48-like + NDM	OXA-48-like + VIM	NDM + VIM	Non CPE	Case	ESBL	ESBL + Case	Imper.	Susceptible to carbapenems^a^
		n	n	n	n	n	n	n	n	n	n	n	n	n	n	n	n
***Klebsiella* spp.**	**1,180**	**633**	**552**	**17**	**51**	**9**	**1**	**0**	**3**	**0**	**0**	**547**	**68**	**338**	**24**	**113**	**4**
***Escherichia coli***	**490**	**256**	**220**	**1**	**28**	**2**	**0**	**0**	**4**	**0**	**1**	**234**	**52**	**118**	**17**	**45**	**2**
***Enterobacter* spp.**	**1,073**	**123**	**101**	**1**	**6**	**9**	**1**	**3**	**0**	**2**	**0**	**950**	**585**	**18**	**326**	**21**	**0**
***Citrobacter* spp.**	**139**	**48**	**39**	**0**	**1**	**7**	**1**	**0**	**0**	**0**	**0**	**91**	**59**	**2**	**26**	**4**	**0**
***Serratia* spp.**	**25**	**7**	**5**	**0**	**0**	**2**	**0**	**0**	**0**	**0**	**0**	**18**	**10**	**2**	**0**	**5**	**1**
**Other species**	**65**	**8**	**3**	**0**	**5**	**0**	**0**	**0**	**0**	**0**	**0**	**57**	**22**	**6**	**3**	**22**	**4**

Case: cephalosporinase; CPE: carbapenemase-producing Enterobacteriaceae; ESBL: extended spectrum beta-lactamase; Iperm: impermeability.

^a^ Susceptible isolates after confirmation of minimum inhibitory concentration values [13].

However, the number of carbapenemase-producers among carbapenem non susceptible *E. coli* isolates rose from 28.2 to 51.8% from 2012 to 2014. Of note, 46 patients were colonised with multiple CPE isolates (representing 106 isolates). In 93.5% (99/106) of the cases, the OXA-48 carbapenemase was identified reflecting the well-known de-repressed transfer properties of the incL/M OXA-48 prototype plasmid [15].

### Mechanisms of decreased susceptibility to carbapenems in non-carbapenemase-producing Enterobacteriaceae

In the absence of carbapenemase production, the decreased susceptibility to carbapenems was mostly explained by a decreased outer-membrane permeability associated with the expression of an ESBL in *K. pneumoniae* (61.8%) and *E. coli* (50.4%). Overexpression of a chromosome-encoded cephalosporinase was mainly involved in natural producers of cephalosporinase that were *Enterobacter* spp. (61.6%), *Citrobacter* spp. (64.8%) and *Serratia* spp. (55.6%) (Table 3). Of note, for these non-carbapenemase producers, decreased susceptibility to ertapenem but retained susceptibility to imipenem and meropenem is frequently observed.

### Colonisation vs infection with carbapenemase-producing Enterobacteriaceae

Among the 1,075 CPE identified in 2014, 643 (59.8%) were from rectal swabs i.e. colonisation and 377 (35.1%) from infection samples (Table 4). These two ratios remained the same since 2012 and were identical regardless of the carbapenemase type [12]. The most frequent clinical samples were urinary samples (68.2%) (Table 4).

**Table 4:** Distribution of specimens from which carbapenemase-producing Enterobacteriaceae were identified, France, 2014

Carbapenemase	Samples from which carbapenemase-producing Enterobacteriaceae were recovered:									Total
	Screening samples (Colonisation)	Infections samples							Information not available	
		Urine	Blood	Respiratory tract	Abscess	Wound	Other	Total infections		
OXA-48-like	556	224	20	29	8	17	24	322	42	920
KPC	12	1	1	2	1	0	1	6	1	19
NDM	50	24	3	1	0	3	5	36	5	91
VIM	15	7	0	2	0	0	2	11	3	29
IMP	1	1	0	0	0	0	0	1	1	3
IMI	3	0	0	0	0	0	0	0	0	3
Multiple carbapenemases	6	0	1	0	0	0	0	1	3	10
Total	643	257	25	34	9	20	32	377	55	1,075

### Carbapenemase-producing Enterobacteriaceae colonisation and travel abroad

**Figure 4:**
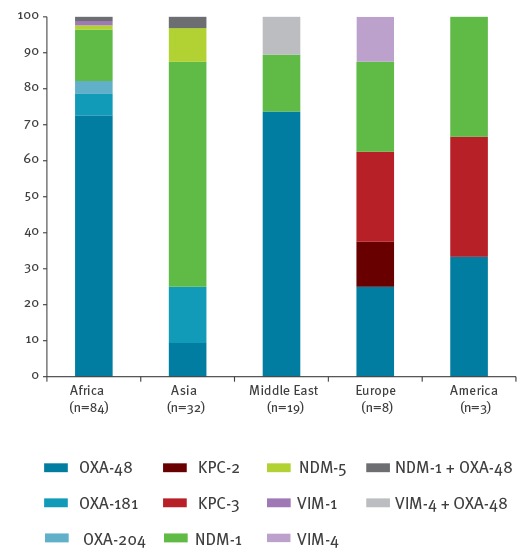
Known geographic origin of possible acquisition of infections and/or colonisations with carbapenemase-producing Enterobacteriaceae, France, 2014 (n=140)

Although epidemiological data were sometimes not well documented, a possible importation from abroad was established for 13.2% (140/1,075) of patients colonised or infected with a CPE in 2014, (27.6% (94/341) in 2012 and 22.8% (145/636) in 2013 (Figure 4).

Only patients for whom travel to a foreign country was known, were included.

**Figure 5:**
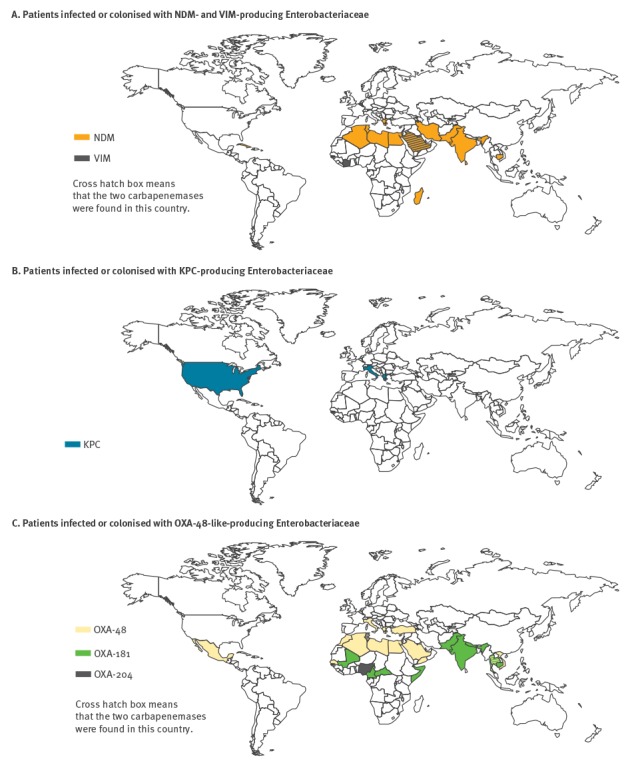
A. Known geographic origin of possible acquisition of infections or colonisation with NDM- and VIM-producing Enterobacteriaceae (n=44) B. KPC-producing Enterobacteriaceae (n=4) C. OXA-48-like- producing Enterobacteriaceae (n=94), France, 2014

From 2012 to 2014, the identification of NDM-producing isolates was often linked to the Indian sub-continent (2012: 17/21; 2013: 17/30, 2014:23/42) where NDM-producers are endemic [12]. In addition, identification of NDM-producers was also observed with travel association from North African (n = 13) and Middle Eastern countries (n = 3) (Figure 5A. KPC producers for which data were available (n=3) were mostly recovered from patients previously hospitalised in endemic countries for KPCs such as Greece (n = 1), Italy (n = 2) and the United States (US) (n = 1) (Figure 5B) [3]. Finally when a link with a foreign country was established (10.2% (n = 94) of the cases for OXA-48-like), OXA-48-like producers were mostly recovered from patients with travel history to Africa and the Middle East (Figure 5C) corresponding to the known spread those CPE in these regions. The 52 OXA-48 variants, and mostly OXA-181 variants (n = 29), were identified from patients returning from the Indian subcontinent (n = 3), South-Eastern Asia (n = 2), and from the tropical region of Africa (n = 5) (Figure 5C).

## Discussion

Carbapenem-resistance in enterobacterial isolates from France were rising about twofold over a three year period with a growing impact of CPE among the Enterobacteriaceae with decreased susceptibility to carbapenems. The increased number of identified CPE mirrored the increasing number of reported nosocomial outbreaks due to CPE in France [16]. However, the resistant isolates were sent by French laboratories to the NRC on a voluntary basis, making their exact prevalence rate unpredictable. For 80% of CPE episodes, sporadic cases or several cases related by an identified chain of transmission, documented by the French Public Health Agency, one or more isolates were characterised by the NRC [17].

The analysis of the 6,682 strains with decreased susceptibility to carbapenems between 2012 and 2014 highlights several features. The three main species with a decreased susceptibility to carbapenems were *K. pneumoniae*, *Enterobacter* spp. and *E. coli*. When compared to 2012, the number of CPE identified in *E. coli* was five times higher in 2014 [12]. This observation hints towards a possible future endemic spread of carbapenemase-producing *E. coli* in the community as previously observed for ESBL-producing *E. coli*.

Overall, the main carbapenemase type identified was OXA-48 as observed in several countries in western Europe (e.g. Belgium, France, Spain) [6]. We suggest that the OXA-48 dissemination is likely the result of strong relationships and population movement between North African countries, where OXA-48 producers are endemic, and France or Belgium and Spain [18-22]. A growing diversity of OXA-48-like variants was identified with OXA-181 most frequently reported. Although the occurrence of OXA-181 is known in the Indian subcontinent and South-Eastern Asia, colonisation with OXA-181 producers in the tropical region of Africa might be more important than expected.

The spread of OXA-181 producers might have previously been missed since one of the most widespread, for example in France, molecular commercial assay for the screening of CPE named Xpert Carba-R performed on the GeneXpert (Cepheid, Sunnyvale, CA, US), did not detect OXA-181 and OXA-232 variants until recently [23,24]. This failure was corrected in the 2015 version of the test that is now available on the market [25,26]. This example underlines that CPE screening should not be limited to molecular tests. Several tests were recently developed for the detection of carbapenem hydrolysis activity such as (i) biochemical test (the Carba NP test and its derivatives RAPIDEC Carba NP, Rapid CARB Screen, blue Carba) [10,27,28], (ii) MALDI-TOF based techniques [29], as well as electrochemical assays (the BYG test) [30]. In addition, in the context of such high prevalence of OXA-48 (ca 80% of the total CPE), rapid immunochromatographic tests able to detect all known OXA-48-like carbapenemases (OXA-48 K-SeTs from Coris BioConcept, BioRad), might be of interest [31,32].

Although the origin of colonisation with a CPE producer was not always documented, it is likely that acquisition abroad is fuelling the growing number of CPE identified in France.

Taken together, our results may indicate that the spread of OXA-48 like and NDM-like producers may soon become difficult to control due to their silent spread in community-acquired *E. coli* as suggested as early as in 2012 [33,34]. Contrary to this, spread of KPC producers that were still identified mostly in *K. pneumoniae*, remained confined to nosocomial settings and should thus still be largely controllable. As exemplified in the public hospitals in Paris (AP-HP), a large regional multi-hospital institution, prevention of outbreaks due to CPE may remain possible when CPE is still mostly a nosocomial problem [16]. Based on our own experience and the results of this study, we advocate for a systematic screening of at-risk patients to identify carriers of CPE. Early screening of patients colonised with CPE is the pre-requisite for the rapid implementation of strict hygiene measures based on isolation of colonised patients and cohorting to prevent and control nosocomial outbreaks.
